# Mycophenolate Mofetil-Induced Colonic Injury Manifesting Endoscopically As Ischemic Colitis

**DOI:** 10.7759/cureus.38856

**Published:** 2023-05-10

**Authors:** Clive J Miranda, Murad H Ali, Muddasir Ayaz, Raheel M Khan, Mayada Ismail

**Affiliations:** 1 Internal Medicine, University at Buffalo, Buffalo, USA; 2 Gastroenterology, University at Buffalo, Buffalo, USA

**Keywords:** colonoscopy, medication side effect, ischemic colitis, immunosuppression, mycophenolate mofetil colitis

## Abstract

Mycophenolate mofetil (MMOF) is a commonly used immunosuppressive prodrug in kidney transplant patients. However, it is not without side effects. The most common of these is diarrhea which inadvertently leads to colonoscopic and endoscopic evaluation when all other workup returns negative. Colonoscopies often show diffuse ulcers and colitis changes depending on the degree of diarrhea. In rare situations, MMOF-induced ischemic colitis may occur on gross endoscopy. We describe an unusual phenomenon of an adult male status post renal transplant with histopathologically diagnosed MMOF-induced colitis who developed gross endoscopic findings concerning ischemic colitis. Our case highlights the importance of recognizing that MMOF-induced colonic changes can rarely mimic ischemic colitis. With this in mind, we aim for gastroenterologists to better understand the varying endoscopic colonic findings of this immunosuppressive drug.

## Introduction

Mycophenolate mofetil (MMOF), an immunosuppressive pro-drug used to prevent solid organ transplant rejection, is hepatically metabolized to mycophenolic acid. This acid reversibly inhibits inosine monophosphate dehydrogenase, which prevents B and T lymphocyte proliferation and leads to leukocyte cytostasis [1-3], as seen in solid organ transplant recipients. Diarrhea is a common gastrointestinal MMOF-induced side effect affecting up to 40% of kidney transplant patients [4]. When all noninvasive workup returns negative, colonoscopies are performed, which often show edematous, erythematous ulcers and diffuse colitis changes. Literature review reports note that colonic histopathology from patients on MMOF sometimes mimics graft-versus-host disease, inflammatory bowel disease, or ischemic colitis. However, very few cases describe segmental colitis in MMOF patients [2,5]. We present a rare case of a kidney transplant patient presenting with MMOF-induced segmental colitis masquerading as ischemic colitis on esophagogastroduodenoscopy (EGD) after four years of medication usage.

This case was previously presented at a meeting abstract at Crohn's and Colitis Congress in February 2023.

## Case presentation

**Figure 1:**
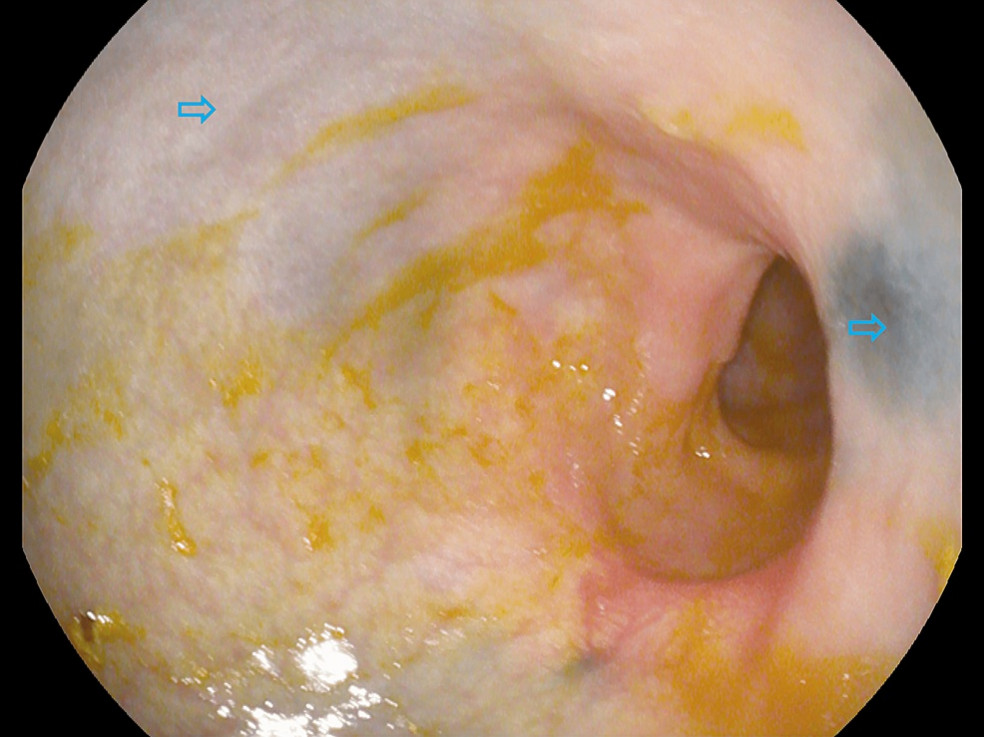
Segmental colitis with punctate erythema and loss of vascularity up to the splenic flexure (arrows) with the distribution of mucosal disruption suggestive of ischemic colitis.

**Figure 2:**
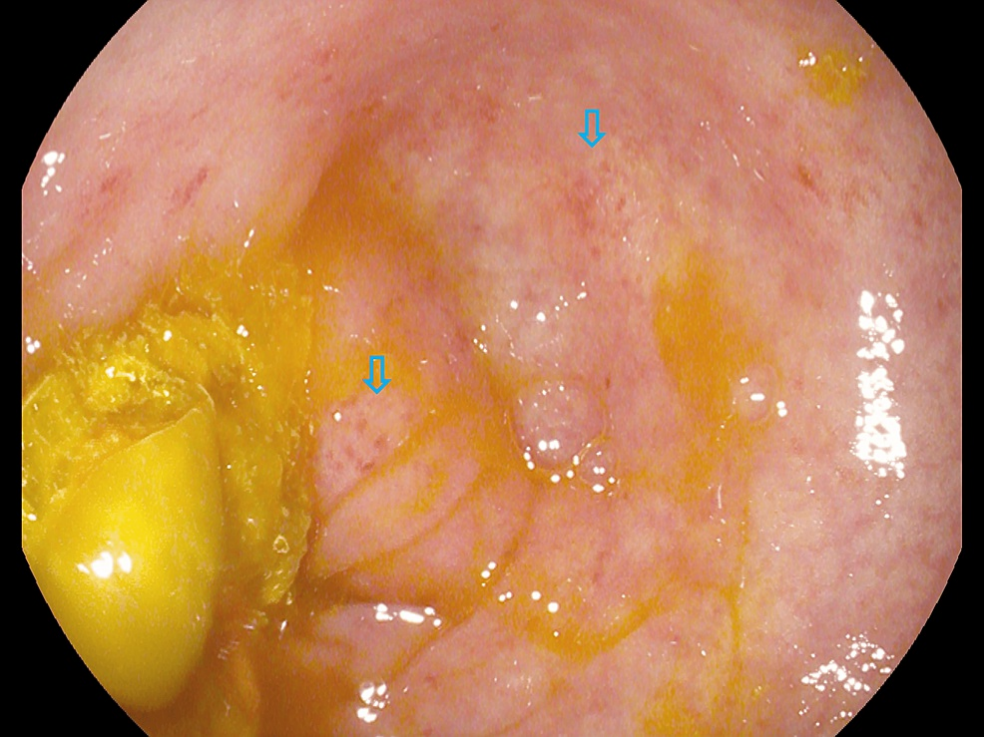
Further evidence of segmental colitis with punctate erythema (arrows) and loss of vascularity up to the splenic flexure with the distribution of mucosal disruption suggestive of ischemic colitis.

A 52-year-old male with a history of end-stage renal disease due to diabetic nephrosclerosis, who received a living-unrelated kidney transplant from his wife four years ago, presented to the ER with one week of intractable abdominal pain, vomiting, and diarrhea. His other medical conditions included type 1 diabetes, coronary artery disease status post coronary artery bypass grafting four years ago, and peripheral vascular disease status post left below-the-knee amputation five years ago. His current transplant medications included atovaquone, tacrolimus 1 mg daily, and mycophenolate mofetil 500 mg daily. Vitals were grossly unremarkable. Lab work showed a leukocyte count of 5.3 x 10^9/L, hemoglobin of 10 g/dL, mean corpuscular volume (MCV) of 85 fL, platelets 58 x 10^9/L, blood urea nitrogen (BUN): Creatinine (Cr) ratio of 87:5.6, and tacrolimus level of 34.8 ng/mL. Inflammatory markers were not elevated. Seven years ago, his last colonoscopy was unremarkable; the patient had never had an EGD. EGD in-house showed significant erosive gastropathy, with pathology showing the same. Sigmoidoscopy showed segmental colitis with punctate erythema and loss of vascularity from the distal colon up to the splenic flexure with the distribution of mucosal disruption suggestive of ischemic colitis (Figures 1-2).

**Figure 3:**
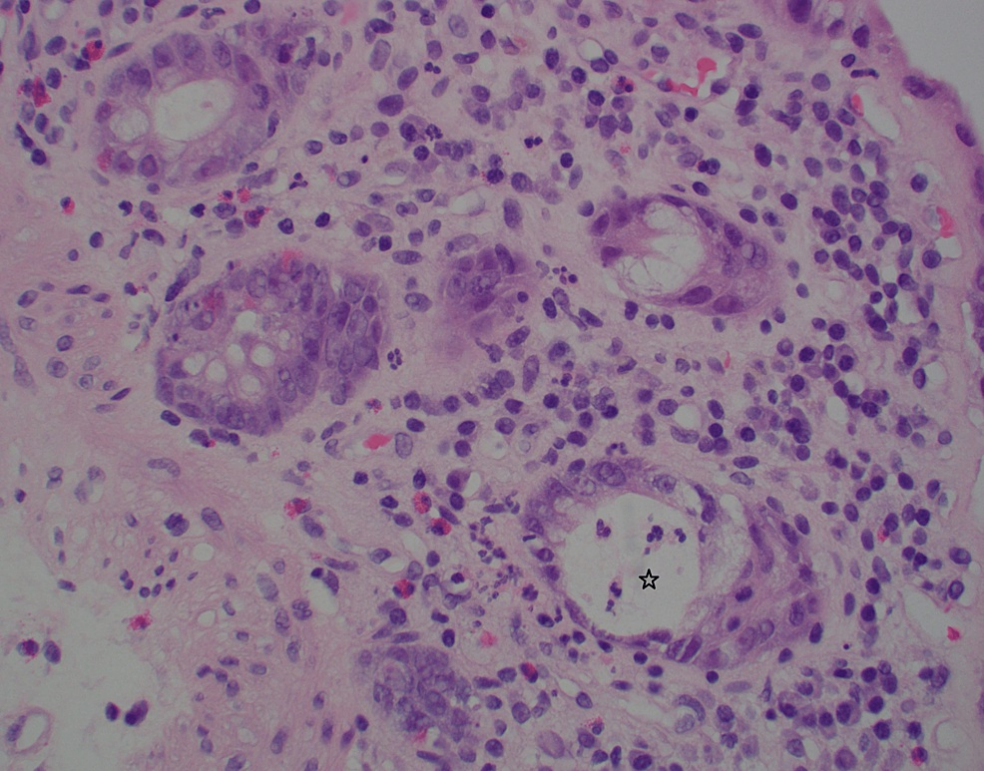
Patchy active cryptitis (star), surface erosion, scattered eosinophils in lamina propria (400x).

**Figure 4:**
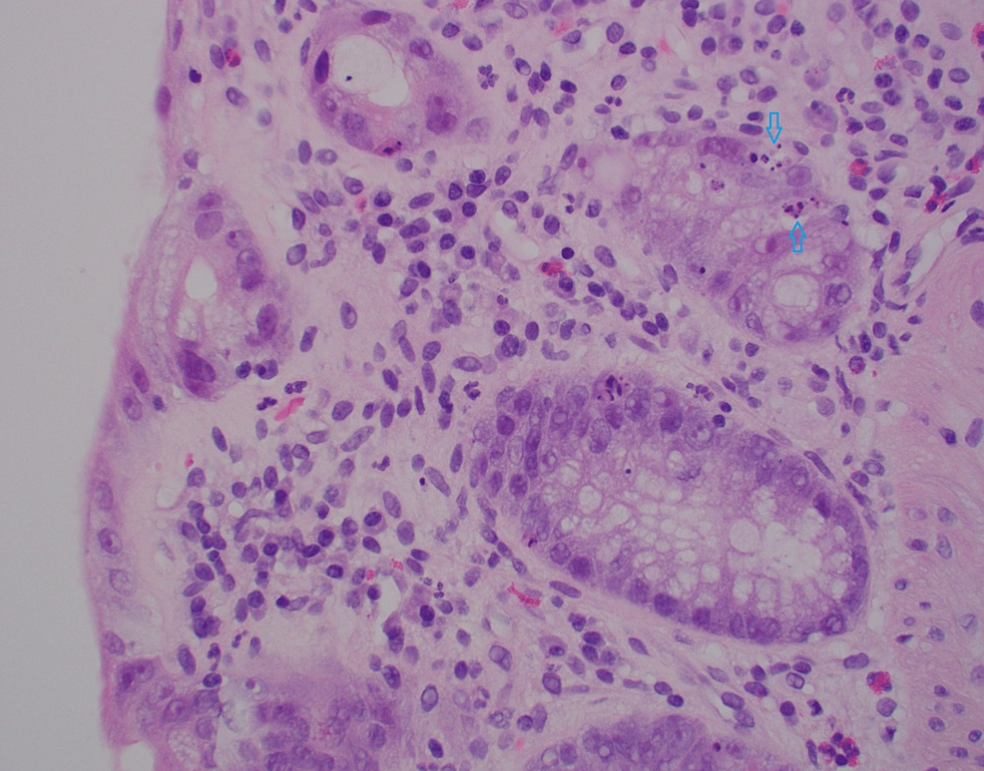
Distorted crypts with concurrent apoptosis (arrow), and eosinophils scattered in lamina propria (400x).

Histopathology showed fragments of colonic mucosa with patchy active cryptitis, surface erosion, distorted crypts with concurrent apoptosis, and scattered eosinophils in the lamina propria. The findings were consistent with mycophenolate colitis (Figures 3-4).

Symptomatic care was provided, and the patient’s mycophenolate was tapered to cessation over two weeks, with complete resolution of his vomiting, diarrhea, and abdominal pain by the tenth day. MMOF was not restarted, and no other new immunosuppressives replaced it. He was discharged with a recommendation for a colonoscopy in 3-6 months to assess the resolution of his colitis. However, the patient was lost to follow-up, so the colonoscopy could not be completed.

## Discussion

MMOF, a commonly used renal transplant immunosuppressant, is not without its side effects, the most common being diarrhea. In patients with long-standing diarrhea and a known usage of MMOF, it is prudent to keep MMOF-induced colitis as a differential diagnosis. Our case highlights the discordance of imaging in colonoscopy and histopathology. While our patient's colonoscopy exhibited findings concerning segmental ischemic colitis, histopathology was negative for ischemia but notable for MMOF-induced injury. Studies suggest symptom resolution occurs between three and 20 days after MMOF therapy is stopped [6-7]. It is believed that mucosal injury from MMOF causes immunotoxicology reactions/inflammation in the colon and decreased mucosal protection. The exact mechanism of this is unclear. Mycophenolic acid, the active form of MMOF, inhibits de novo purine synthesis by blocking inosine monophosphate dehydrogenase, a key enzyme in this synthetic pathway. Enterocytes are 50% dependent on de novo purine synthesis. It is believed that this mechanism, along with acyl glucuronide metabolite-induced toxicity and the antibacterial effect of MMOF, play key roles in the mechanism of MMOF-induced colonic injury [6-8]. Decreased colonic protection leads to disrupted membrane phospholipids, decreased mucosal defense, and the local irritation caused by epithelium-induced mononuclear cells releasing TNF-alpha, causing mucosal inflammation [8].

MMOF-induced colonic injury can endoscopically resemble an array of other conditions, including acute colitis, graft-versus-host disease, ischemia, and inflammatory bowel disease. Mucosal biopsies are thereby essential to differentiate between these conditions [9]. Histopathological features specific to MMF-induced colitis may include but are not limited to, dilated and damaged crypts, crypt architectural distortion, increased inflammation of the lamina propria, increased apoptosis of crypt epithelial cells, and graft-versus-host-like changes [10-11].

There is no consensus on the treatment of MMOF-induced colitis. Some reports advocate discontinuing the offending MMOF agent with self-resolution of symptoms shortly after [11]. Persistent symptoms refractory to MMOF-discontinuation may benefit from the addition of steroid or infliximab treatment.

Our case highlights and urges clinicians to differentiate MMOF-induced injury from ischemic colitis by showing the discordance between endoscopic visualization and histopathology. With early recognition of this phenomenon, patient symptomatology can be markedly improved with subsequent MMOF discontinuation [10].

This case was previously presented as a meeting abstract at the Crohn's and Colitis Congress in February 2023 in Denver, Colorado [12].

## Conclusions

In conclusion, MMOF can have several notable adverse reactions on colonic mucosa, with one of the rarest phenotypes mimicking colonic ischemia. There is currently sparse literature on the condition with several lingering questions on pathophysiology, optimal treatment, and post-treatment monitoring. Often, further problems are manifested by suboptimal communication between gastroenterologists and pathologists as, all too often, pathologists are not informed of the patient's MMOF regimen, which can thereby delay timely diagnosis and effective treatment. Clinicians must take care to note both histopathologic and gross endoscopic findings concurrently. Timely recognition of this condition, with appropriate MMOF removal, can yield a significantly more favorable prognosis for the patient.
